# Taking gender seriously in climate change adaptation and sustainability science research: views from feminist debates and sub-Saharan small-scale agriculture

**DOI:** 10.1007/s11625-017-0464-y

**Published:** 2017-08-19

**Authors:** Anne Jerneck

**Affiliations:** Lund University Centre for Sustainability Studies, Lund University, Box 170, 221 00 Lund, Sweden

**Keywords:** Development, Distribution, Empowerment, Environment, Inequality, Poverty

## Abstract

People, places, and production contributing the least to climate change will suffer the most. This calls for adaptation as a key climate change response. But adaptation is surrounded by problems. Finance is uncertain and fragmented, mainstreaming into development is complicated, and technical solutions often overshadow existing social relations and institutions. From a gender perspective, and as a critical research initiative to support the building of sustainability science as an umbrella field, this article raises three pertinent questions on adaptation in the global South: what is its purpose, how can development inform it, and what institutions in terms of rights and responsibilities are core to it? Focusing on sub-Saharan small-scale agriculture, three main points emerge. Regarding the purpose, adaptation should be a transformative pathway out of poverty, ill-health, and food insecurity. Regarding development, adaptation can learn from how development theory, policy, and practice have addressed women, gender, and environment in varied settings and debates. Regarding core institutions, adaptation must address gender regimes that regulate access to, use of, and control over resources, especially those defining land distribution, labour division, and strategic decision-making power. To conclude, I propose gender-informed research questions for further inquiry.

## Introduction

There is an increasing number of studies on climate change adaptation, but it is still disproportionate to the importance of the issue. Few studies have tested how adaptation is perceived and understood at the nexus of climate change and poverty (Moser 2014). Even fewer studies have explored how exposed settings in rainfed small-scale agriculture in sub-Saharan Africa (SSA) experience climate change and poverty in relation to gender as a core analytical category. Research in sustainability science (SS) has identified key conditions that are crucial for understanding the scope for climate change adaptation and poverty alleviation in this region (Gabrielsson et al. 2013; Jerneck and Olsson 2013; Gabrielsson 2014). The food and health condition imply that small-scale farmers strive to secure food and fend off threats to their health (Gabrielsson et al. 2013; Jerneck and Olsson 2013; Gabrielsson 2014). The gender condition refers to how gender as a higher order process and a fundamental social relation in society is manifested in the gender regimes of land, labour, and marital love (Steen 2011). The regimes institutionalise how resources are accessed, distributed, and consumed, how labour is coded, re-coded, and divided into productive and reproductive tasks, and how social practices and responsibilities are discursively defined and fulfilled. Together these conditions affect how farmers perceive risk, prioritise and share tasks in everyday farming, experience hardship and shape aspirations about future livelihoods. All this will influence the adaptation space. To be successful, climate change adaptation and technology adoption informed by SS must therefore take these conditions into account (Jerneck and Olsson 2014).

The article is outlined along three pertinent questions on climate change adaptation in the global South: what is its purpose, how can similar processes, such as development, inform it, and what institutions in terms of rights and responsibilities are core to it? To address the first question, on the purpose of adaptation, I discuss three key aspects: inequality in the causes, impacts, and responses to climate change (Sect. 2); variation in the definitions, framings, and understandings of climate change adaptation; and adaptation in the midst of multiple stressors and vulnerability in small-scale agriculture (Sect. 3). To justify a discussion on adaptation in the light of development and sustainability, I refer to the often repeated argument that they must be tackled in combination (Klein et al. 2005) partly because of obvious overlaps and synergies but mainly because ‘climate change risks are redefining what development policies can accomplish’ (Agrawal and Lemos 2015). To address the second question, on how adaptation in the global South relates to development, I discuss gender dynamics of poverty, inequality, and distribution in small-scale agriculture (Sect. 4). To address the third question, on core institutions in adaptation, I propose a frame with questions for how one could proceed in gender-informed research on climate change adaptation (Sects. 5, 6). Inspiration comes from three main sources. First, an editorial by Jon Barnett posing similar questions for policy-relevant research on climate change adaptation: what is its purpose, are there analogies, and which institutions matter (Barnett 2010)? Second, sustainability science research on food, gender, and health conditions in sub-Saharan small-scale agriculture, here referred to as the ‘three imperatives’ (Jerneck and Olsson 2014), and third, literature on power asymmetries in the distribution, procedure, and recognition of climate justice in adaptation and development (Popke et al. 2016; Whyte 2014). Speaking of climate change adaptation in *interdisciplinary* terms helps capturing the complexity of natural and social dimensions of human–environment interaction (Jerneck et al. 2011; Moosa and Tuana 2014). In addition, it furthers the use of sustainability science as an umbrella field for integrated knowledge (Shahadu 2016) based on pluralism in theory and methods (Isgren et al. 2017). Below, I define core concepts and explain the rationale for gender-informed research.

### Climate change adaptation

IPCC defines climate change adaptation as ‘adjustment in natural or human systems in response to actual or expected climate stimuli or their effects which moderates harm or exploits beneficial opportunities’ (IPCC 2007). As also confirmed by IPCC (Noble et al. 2014) and others (Leighton et al. 2011), mainstream literature often strips climate change adaptation of its human content and social context while instead linking it to technology, such as climate-proofing of infrastructure, or economic tools, such as insurance policies, with dire consequences for poverty alleviation. Meanwhile, adaptation seen as a process of profound *social* change in livelihood activities, or as *transformation*, gets less attention (Khan and Roberts 2013). That void will be addressed here from the perspective of farming communities in sub-Saharan Africa representing one of the most climate change vulnerable areas in the world (World Bank 2012).

Adaptation and mitigation are socially, spatially, and temporally differentiated responses to climate change. Whereas mitigation refers to reduction of hazard, exposure, and vulnerability to potential adverse impacts of climate change, adaptation means adjusting to actual or expected climate effects. Adaptation can be incremental serving to maintain a system/process, or transformative serving to fundamentally change system attributes. It can be protective in terms of taking preventive measures against negative impacts or opportunistic in terms of taking advantage of potential beneficial effects of climate change (IPCC 2014a, b). Although poor communities in the global South would prioritise adaptation over mitigation, some of the most effective adaptation measures are also important mitigation strategies (Tschakert and Olsson 2005; Olsson and Jerneck 2010) as exemplified by the technology adoption of: agro-forestry (Sanchez 2000), altered agricultural practices (Farage et al. 2007) and improved cooking-stoves consuming less fuelwood while releasing fewer pollutants and lower emissions (Olsson and Jerneck 2010; Jerneck and Olsson 2012). Still, populations who are poor and highly exposed to climatic events may be severely constrained in promoting their own (economic or political) agenda (UNDP 2009). To advance their cause in international climate change negotiation and policies, it is pertinent to demonstrate that this population is big, their potential contribution to mitigation is decisive, and strategies that cement or reinforce existing inequalities should be avoided (Olsson and Jerneck 2010).

### What about gender in climate change adaptation?

Farming livelihoods in small-scale agriculture are imbued by gendered divisions of rights and responsibilities (Doss 2001) expressed in power asymmetries in access to land, labour, and leisure time. This social differentiation may imply varied vulnerability and capacity to adapt to climate change, climate variability, and other stressors (Vincent et al. 2014). However, it should be noted that while some social norms and relations seem fixed, others are fluid and flexible (Farnworth and Colverson 2015), especially in times of social change.

Given that climate change adaptation entails structural and intersectional power, critical geographers argue that research and policy must disentangle social processes and practices and be sensitive to intersecting inequalities that emerge when climate change impacts and responses cut across age, class, ethnicity, gender, and space (Sultana 2014). For that purpose, gender-informed approaches, and disaggregated data are essential (Alston 2014). Counting, measuring, and mapping may provide correlations and quantitative overviews for comparison, whereas in-depth research on social structures and individual practices in everyday life is needed for understanding causality (Jönsson et al. 2012). Since available evidence is ‘limited, patchy, varied and highly contextual’ it is not yet widely confirmed that/how climate change has differential impact on women and men in terms of assets, agency, and achievements, but data are fairly consistent with the two propositions that ‘climate impacts may affect men and women differently’ and ‘women tend to suffer more negatively in terms of their assets and well-being’ (Goh 2012). This vulnerability, as documented by several studies, is not intrinsic to women but due to the gendered distribution and organisation of land and labour (Pearse 2017).

According to the WHO, women suffer higher risk than men in health and life expectancy and are harder hit by floods, heavy rains, heatwaves, drought, and water scarcity (WHO 2014) often associated with climate change. Besides, women have less access to critical information on cropping patterns and weather alerts (WHO 2014). Due to likely ‘uneven impacts of climate change on women’ (Alston 2013) there is need for critical feminist research that not only describes but also explains such gender asymmetries (Pearse 2017). In so doing it should consider women and men as equal partners facing environmental change and, consequently, it should suggest policies to promote socio-environmental justice (Israel and Sachs 2013).

Despite the fact that adaptation is gendered and varies across scales and subjectivities (Sultana 2014), knowledge on how climate change interacts with geographical and social inequality is underrepresented in policy and research (Dankelman 2010; Hammond 2012; Olsson et al. 2014). In a bibliometric analysis using the ISI Web of Science to search abstracts, keywords, and titles for the terms ‘climate’ AND ‘adaptation’ AND ‘gender’ in research published January 2000–April 2017, I found 188 social science articles containing the three concepts. Few of these (only 17) were published before 2011. After that, the number of articles and the citations they receive have grown exponentially. In comparison, and using the same approach, I found 7004 social science articles on ‘climate’ AND ‘adaptation’ published January 2000–April 2017. This search in the ISI database is only a rough indication. It does not exclude that there are other research articles relevant for climate change adaptation and gender. To exemplify, the cross-chapter box on gender and climate in IPCC AR5 refers to 34 articles, of which only every other title overlaps with my search (Vincent et al. 2014). Now that the agenda on women–environment dynamics is gaining strength from the climate change debate more gender relevant research is on its way (Resurrección 2013). And although empowerment of women in relation to climate change policy varies between countries, there are signs that the mainstreaming of gender and women’s needs in climate change responses is increasing in policy documents in East and Southern Africa (Nhamo 2014, p 156). Nonetheless, it is evident that gender is under-researched within climate change adaptation. Explorative and systematic studies on power and ethics (Moosa and Tuana 2014), distribution and environmental justice (Jerneck 2015), and impacts and responses (Pearse 2017) would thus contribute further valuable knowledge.

As regards policy and practice, the number of documented adaptation initiatives has increased significantly since 2006 albeit from a low level, especially in Africa, particularly in Kenya, and mainly in semi-arid agriculture (Ford et al. 2015). An analysis of these initiatives indicates that most of them are national rather than local and cover both pro- and reactive responses while few of them consider vulnerable groups or show evidence of substantive adaptation (Ford et al. 2015). To conclude here, the brief overview above serves as a rationale for doing (more) gender-informed research on climate change adaptation.

## The significance of climate change, inequality, and poverty

Climate change is a formidable challenge. It entails a combination of slow-onset and rapid-onset events with uncertain and uneven impacts (Field et al. 2014) and is expected to become more frequent and forceful, especially in sub-Saharan Africa (Lobell et al. 2008; World Bank 2012; Field et al. 2014). It is accelerating to the extent that the risk of failing to stabilise the climate at the 2-degrees-target is real, making both adaptation and mitigation more urgent (Stocker 2013; Geden and Beck 2014; Stocker 2014). There is a risk, though, of reaching limits to adaptation especially in places that are most exposed to climate change impacts such as sub-Saharan agriculture where profound social change, rather than merely adjustment, should be a main purpose of adaptation (Noble et al. 2014). However, this is not without problems, neither in terms of who gets to decide the direction or the depth of that transformation nor its distributional outcome. Once the difficulties of transformational adaptation are recognised it is necessary to discuss how to grasp potential opportunities while remembering that adaptation is ‘underpinned by diverse values’ varying across contexts and cultures (Adger et al. 2009, 2013a).

### Spatial, cultural, and temporal inequality

Climate change is transboundary and requires multi-scalar agreements, efforts, and solutions. In comparison to other environmental debates, the climate change debate has therefore been less localised, community-based, or centred on agency, identity, and gender (Leach 2007). But with strong and increasing evidence that frequent droughts, intense heat waves, and serious flooding will be socially and spatially differentiated (World Bank 2012; IPCC 2014a, b) the global focus must shift towards practical policies in the local where variations in real experiences, capacities, and initiatives emerge. Importantly, complexity increases dramatically when the focus is shifted from global to local settings where climate change impacts and effects intersect with major social forces such as commodification, marketisation, technological shifts or large-scale social intervention in the environment (O’Brien and Wolf 2010) while also being embedded in and mediated by culture (Adger et al. 2013b).

Income-generating resources and livelihood opportunities are impacted by climate change mainly because they are governed by differentiated decision-making power, institutions and social relations (De Haan and Zoomers 2005). In the global South, where most people who are poor reside in rural areas and rely on agriculture for their living and livelihoods (Dercon 2009) climate change will have major impacts not only on availability and distribution, but also on destruction of resources, including agricultural land, and all these processes may in themselves further reinforce social differentiation (World Bank 2012). In rural areas those who have limited access and rights to resources, while being responsible for food production, will be the most vulnerable to impacts of climate change and climate variability (UNDP 2007). Gender-informed data show that climate change will have disproportionate effects on small-scale farmers, many of whom are women in sub-Saharan agriculture who depend for their living on degrading physical resources—land, water, forest products—and who are already under pressure from the multiple stressors of poverty, ill-health, and food insecurity (UNDP 2009; Hammond 2012; World Bank 2012) as also confirmed in research on the food, health, and gender imperatives (Jerneck and Olsson 2014).

Beyond social and spatial differentiation, there are temporal aspects. In sub-Saharan Africa, where climate change already has an impact on natural resources and rural livelihoods due to recurring floods and droughts (World Bank 2012; Olsson et al. 2014), the availability of systematic multi-scalar knowledge on adaptation to and mitigation of climate change may help farmers in the short run to adapt their livelihoods while also informing policy on human security and wellbeing. But if short-term change, such as introducing new specific practices or asserting well-known strategies, blocks or postpones necessary long-term change this may in fact result in mal-adaptation rather than adaptation (Brooks et al. 2009, p 741). Ill-considered efforts to improve ‘wellbeing’ or protect ‘human security’ may thus end up masking inequality (Hudson 2005) rather than tackling the underlying reasons for the need to adapt (Inderberg et al. 2014).

## The purpose of adaptation and the importance of context

Adaptation is widely recognised as one of two main response options to reduce the risks from climate change. It involves established major practices such as agricultural outreach, coastal management, disaster risk management, resource management, spatial and urban planning, and public health (Füssel 2007, p 268). It refers to the ability of individuals, societies, and systems to cope with multi-scalar processes (Dodman and Mitlin 2013) and to use information on present and future climate change in relation to the suitability of current and planned policies, practices and infrastructure (Füssel 2007, p 268). It has to be informed not only by geographical insights on bio-physical, geo-morphological, and hydrological conditions, but also by socio-technological conditions and relations. As risks cannot be fully eliminated, adaptation needs to reduce exposure and vulnerability while also increasing capacity to resist or recover from the potential adverse impacts of climate extremes (Field et al. 2012).

Despite substantial investments in research, following from the mounting need for adaptation, practical progress is slow, partly because of the way in which adaptation is framed and understood (Ford et al. 2015). Difficulties also arise from the fact that adaptation is inseparable from its socio-ecological context, and, just like development, highly influenced by historical conditions and properties emerging in the cause of action (Wise et al. 2014). Climate change adaptation in a particular setting should thus be prepared through ‘ground work actions’ followed by ‘concrete actions’ including the implementation of institutional guidelines and public awareness (Ford et al. 2015).

Hence, adaptation must involve adjustments to current activities or imply fundamental change and social transformation that needs to be negotiated (Field et al. 2012, p 3). This makes adaptation a contested and power-laden process of ideas and interactions at multiple scales influencing existing social differentiation and inequality (Nightingale 2009).

### Adaptation as adjustment, development reform, or transformation

Adaptation is closely associated with climate change vulnerability and how we speak of their interdependence will influence how we act (Bassett and Fogelman 2013). IPCC sees climate impacts as a main source of vulnerability which calls for adaptation as adjustment; others locate risks in both nature and society as a source of vulnerability thus seeing adaptation as social reform or development and a means to reduce vulnerability within prevailing systems; but little research explores the (deeper) social drivers of vulnerability or the need to understand and achieve adaptation as a political-economic transformation (Bassett and Fogelman 2013) and as *transformative* climate action (Pelling and Manuel-Navarrete 2011; Ribot 2011).

Although major social processes interact and overlap, many development agencies and practitioners distinguish (too much) between climate change adaptation, disaster risk reduction, and poverty alleviation (Reid and Schipper 2014). In the global South, underlying ideological and theoretical assumptions about development may influence interpretations of adaptation and vulnerability (Ireland and McKinnon 2013). Using adaptation to reconstitute conventional growth-driven development is not necessarily acceptable, efficient or fair, particularly not from post-development perspectives seeking to shift the focus towards climate change drivers and the aspirations of local communities (Ireland and McKinnon 2013). Using a general recipe for adaptation is not necessarily the most productive way to address climate change and donors must ‘refuse to know exactly what should be done or how’ (Ireland and McKinnon 2013). Instead, adaptation could be an opportunity to go beyond conventional development and seek to implement the unfulfilled promises to reduce poverty and inequality while enhancing sustainability (Jerneck and Olsson 2008).

### Adaptation and vulnerability as gendered phenomena

There is no single best definition of vulnerability because context and purpose are important for assessing it (Füssel 2007). Often it is seen as intertwined with poverty, but plays out differently depending on the social and political ecology of the context. In the gender literature it has been argued that vulnerability is neither a fixed nor an intrinsic characteristic of certain people or groups of people (Enarson 1998) or derived from a single social dimension like being poor, rural, woman, or part of a particular (or marginalised) community (Blaikie et al. 2014). Rather, it depends on social and historical ‘patterns of practices, processes and power relations that render some groups or persons more disadvantaged than others’ (Enarson 1998) and more vulnerable to risk and disaster than others (Hilhorst and Bankoff 2004). If vulnerability must cover not only differentiated and changing circumstances before, during, and after a disaster or hazard (Blaikie et al. 2014), but also historically and culturally determined conditions (Enarson 1998), then it is less an individual or personal feature and more a structural, relational, and process-oriented condition.

Climate change impacts and responses interact with existing and emerging gendered capacities and vulnerabilities in complex ways, often resulting in unintended consequences (UNDP 2010, p 39). To take an example, the loss of assets, crops, gardens, home-based production, and livestock in consequence of water-related hazards such as cyclones, droughts, erosion, floods, and land-slides may all have gendered implications. Women and men may be affected differently, depending on how social relations shape rights and responsibilities in production, reproduction, and decision-making (Alston 2013). And if women are more vulnerable to climate change impacts they may also be disadvantaged in access to adaptation resources (Demetriades and Esplen 2008; Ford et al. 2015). Research on the effects on equity, efficiency, and identity will thus necessitate further studies on the gender dynamics of climate change (O’Neill et al. 2010).

Despite specific variations, adaptive capacity as a response to climate change impacts, and a means to reduce vulnerability, can be defined as the general ability to anticipate, absorb, accommodate to or recover from the effects of extreme events (International Strategy for Disaster Reduction 2004). Adaptive capacity for women and men to reduce their respective vulnerability in a given context may depend significantly on underlying norms that define and regulate the use of space and the power associated with that (Jabeen 2014). Situated knowledge and social reproduction are therefore useful concepts to analyse how social and spatial locations shape everyday activities and practices and how that relates to what wo/men know and how they respond to social and environmental stressors (Bee 2013).

### Adaptation in small-scale farming

For a fact, climate events, such as droughts, floods, and changing weather patterns, are already causing problems for climate-dependent livelihoods in small-scale farming and livestock-rearing. In the absence of stabilisation, climate change will exacerbate such problems in the coming decades, especially in southern Africa where projections consistently predict decreasing rainfall, intensified droughts, and increasing variability (Solomon et al. 2009; World Bank 2012; IPCC 2013, 2014a, b). Agriculture, biodiversity, ecosystems, water resources, and human health will be adversely affected (World Bank 2012), while seasonal shifts and climate events such as droughts, floods, and storms will be increasingly unpredictable and more intense (Field et al. 2012). But due to uneven and uncertain impacts it is hard to decide—in a certain area—what is attributable to climate change ***or*** what to other conditions (Cramer et al. 2014). Hence, climate change must be understood in relation to the core stressors that populations experience in everyday life, such as land-use change, food insecurity, and ill-health in the context of persistent poverty (Jerneck et al. 2011; Jerneck and Olsson 2015).

For local settings, climate change observers and development practitioners often suggest community-based adaptation on the premise that communities have the necessary expertise and networks to initiate appropriate activities to avoid loss or speed up recovery (Dodman and Mitlin 2013, p 2). Local knowledge for action is indeed important, but there are conceptual, methodological, and political problems with community-based approaches to climate change, one of which is the common focus on a *single* challenge (climate change) rather than on the multiple interlocking aspects of vulnerability (Dodman and Mitlin 2013, p 3–6). A focus on the local only, at the expense of the national or global level, can also be problematic (Adger et al. 2005) because it may marginalise or put disproportionate pressure on the local. In addition, the tendency to assume that the ‘community’ is a uniform and inclusive entity may underestimate power, inequality, and diverging interests within it. Nonetheless, research shows that although it is time consuming to assess the merits or limits of community-based adaptation, valuable lessons can be drawn from good initiatives that may potentially be either scaled up (Reid and Schipper 2014) or scaled out.

To sum up on the first question, adaptation may seem clear in purpose, aiming both for adjustment and transformation, while it is evidently difficult in implementation given that climate change impacts are inherently unequal and power-laden and that adaptation as a response entails conflicting multi-scalar views on everything from resource use to the degree and direction of social change. The second question will focus on what can be learned from development, especially on gender, as a major process with implications for adaptation.

## Learning from development dynamics: gender, power, and environment

The social construct of gender shapes social relations in profound ways. Doing gender means to be guided by norms and rules in ‘perceptual, interactional and micro-political activities’ that are ‘embedded in everyday interaction’ (West and Zimmerman 1987, p 125–126). As a higher order process in society and of particular importance here as an analytical category, gender in small-scale agriculture determines the division of rights, responsibilities, and risks in relation to the use, management of, and control over environmental resources, especially land.

Feminist literature has noticed that the duality of seeing ‘women’ as either vulnerable victims or competent agents and virtues (Arora-Jonsson 2011)—or both (Chan 2014, p 6953)—is repeatedly stressed in debates on environment–development (Leach 2007), migration–development (Jackson 1993; Piper 2009; Chan 2014), and security and peace-building (Cohn 2008). Feminists have observed that in everyday practices of how to use space and time, gender is mediated in dynamic processes giving rise to ‘separate spheres’ of varying priorities and privileges such as the public versus the private realm. Within the private realm gendered space is often separated further into areas for cooking, living, and working (Jabeen 2014) and although gender norms may appear as fixed—reproductive and productive labour are constantly reinterpreted in both theory and practice (Reed and Mitchell 2003). This means that owing to institutional instability and fluidity in norms, gender is subject to ongoing socio-cultural negotiations whereby women with intra-household bargaining power become resourceful agents of change (Cruz-Torres and McElwee 2012; Doss 2013).

How best to use analytical categories suggested by feminist theory is debated. In the development discourse, the notion of ‘woman/women’ as a concrete singular category has often been centre stage, for good or for bad, at the cost of femininity/masculinity or gender as relational concepts. Early works in development put ‘woman/women’ in focus to highlight precarious conditions relating to health, labour, social status, and work load whereas later debates have often referred to women in their capacity as capable family breadwinners, care-takers, and entrepreneurs (Jerneck 2015). This has served the (good) purpose of making women visible in gendered production and reproduction, but if men and masculinity are thereby constantly made invisible in reproduction then *relational* aspects of gender are lost and understandings of society and culture ultimately become partial or even distorted.

Inspired by development discourses that see gender, inequality, and poverty as theoretically and empirically intertwined, and given the relevance of that for climate justice in a warmer world, I suggest seven condensed development themes as an analogy for how to think about and understand gender and sustainability in climate change adaptation. The intention is not to include every aspect of and all contributors to these debates, but to characterise them for the sake of learning lessons. In all, they refer to how women are socially represented, to what extent their rights are recognised, and whether resources can be fairly redistributed (Fraser 2009). Starting historically, I move towards contemporary debates.

‘*Short-lived historical gains*’: Throughout history, social and cultural norms have been contested, challenged and changed especially during major technological transformation or social upheaval such as agrarian reform or revolution, civil conflict or international war, economic boom or crisis, natural disaster or, as of late, climate change events. Asia exhibits many well-known historical examples of how gender is constructed and contingent but also fluid and flexible in the course of decisive economic or political moments (Jerneck 2015). Despite their strategic gains in the public sphere during these events, women who entered wage-work or administrative positions in war time often remained in charge of reproductive household work, seemingly more resistant to recoding and change (Resurreccion 2011). Gains in terms of social recoding of gendered positions and relations appeared fragile because gender soon reverted to ‘normal’ in the aftermath of a major event during which gender norms and social relations were recoded and changed in unexpected and exceptional ways.

‘*Integrating women but ignoring gender*’: Ester Boserup (1970), who was an early proponent of gender equality in modernisation theory, argued that women should be further integrated into the economy to reap benefits from development. In contrast, radical feminist scholars stated that women were not only already involved but also exploited in both reproductive and productive work (Beneria and Sen 1981). Concerns about women’s low income, heavy workload, and ‘time poverty’ have since then been mainstreamed into development thinking (Blackden and Wodon 2006). But interventions that build on the idea of women’s integration and which seek to involve women in the public sphere often seriously overlook the role of men and masculinity in the economy, in society, and in the private sphere thus ignoring that gender is relational (Krishna 2012, p 15) only to end up with limited understandings of society and its fundamentally relational structures and interactions.

‘*Equity, efficiency, and identity*’: Gender has been discussed from many angles of development such as equality and fairness in access to socio-economic opportunities, exclusion or integration, exploitation or empowerment, marginalisation or participation, and also in terms of equity as an inclusive means to strengthen development effectiveness (Tinker 1990). With climate change it is an increasing challenge for policy and research that on top of all aspirations and expectations associated with development (UNDP 2010, p 39) adaptation must consider both existing gender inequalities and emerging gendered vulnerabilities. From a feminist perspective, the dynamics of climate change impacts and responses in the context of multiple stressors should thus explicitly consider the gendered and intersectional effects on equality, efficiency, and identity (Terry 2009; Vincent et al. 2014).

‘*Rights to resources*’: Gendered agency and institutions create differences in capacities, incentives, and preferences for how to manage resources. They also influence means, motives, and conditions for how to contribute to adaptation and sustainability. Knowledge about the environment can be gendered, as can attitudes and abilities that determine how farmers manage natural resources and approach new farming techniques and practices (Doss 2001) (Farnworth and Colverson 2015). Rights to resources may appear as bundles of private, common or public goods (Schlager and Ostrom 1992) and may differ between customary or statutory legal institutions (Meinzen-Dick et al. 2014). In settings where state-recognised property rights are lacking, gender regimes may instead govern access to, ownership of, and control over resources (Behrman et al. 2012). Rights to resources can be temporary if a certain gendered activity or seasonal agricultural practice is associated with a certain resource (Leach 1992) or permanent if women risk losing their land rights, such as in case of divorce or widowhood (Steen 2011).

‘*Being close (and vulnerable) to nature*’: A recurring argument warns that women run the potential risk of carrying the burden of environmental care and management because they are ‘closer to nature, are hardest hit by environmental degradation, and have special knowledge of natural resource systems’ (Resurrección 2013, p 33). Feminist scholars who criticise such essentialist views in the development debate about the strategic but vulnerable position of women have called for nuanced interpretations of gendered relations to natural resources in agriculture, forestry, and water management (Leach 2007). But the argument is still gaining importance in debates about how global warming influences the women–environment dynamics (Resurrección 2013). According to this reasoning, climate change and the many alterations in natural conditions that follow in its wake (including environmental degradation, land-use change, and water scarcity) will render women even more vulnerable if they are too close to and dependent on nature.

‘*Perceptions, priorities, and expectations*’: In a given setting, women’s and men’s experience of climate change and climate variability may vary, as may also their perceptions of the associated risks, their priorities and perceived skills and responsibilities in relation to adaptation and local gender norms, their gendered responsibilities and differences in decision-making power in relation to environmental resources, and their thoughts about mitigation of climate change (Dankelman 2010). Such differences may be rooted in and driven by gendered livelihood activities and farming practices and in how women and men depend on and act differently in relation to environmental resources owing to varied reproductive responsibilities (Terry 2009; Otzelberger 2011). Yet, these conditions may be malleable.

‘*Neither fair–Nor fixed*’: In the event of human-environmental change, gendered norms in reproduction and production may be challenged. After a climate change event, gender-specific impacts may build on and exacerbate gendered relations and responsibilities, while new obligations arise (OXFAM 2009). As an illustration, (women’s) workloads relating to climate change adaptation and mitigation may increase if new tasks are added on to old ones (Blackden and Wodon 2006). Conversely, work may equalise if labour is re-coded from being feminine to becoming masculine such that men assume women’s tasks and women assume men’s tasks as happened in a severe drought in Cambodia when women and men reinterpreted, shifted and shared work tasks in agriculture to overcome shared difficulties (OXFAM 2009; Resurreccion 2011). A crisis may reinforce gender-based disadvantages, but may also entail negotiations and change in seemingly fixed gender relations, thereby in part destabilising gender. In sub-Saharan small-scale farming it has long been noted that changes in the gendering of tasks occur when men exploit new opportunities outside agriculture and women expand their working repertoire in food production—however, men take up women’s tasks (only) when they become profitable (Doss 2001; Farnworth and Colverson 2015).

### Learning from an analogy with development

In response to the second question, I discussed multiple gender aspects of development defined as a major social process comparable to and intertwined with adaptation. I now move on to the third question on the core institutions that are critical to climate change adaptation.

## Core institutions: access to and control of social, material, and discursive resources

Both historical and contemporary processes determine how climate change impacts are experienced and acted upon by regions, communities, and individuals, and how adaptation will be initiated, managed, and governed. In areas where people who are poor depend on agriculture for their living the key to poverty alleviation is to have access to income-generating resources, whereas the key to adaptation is the ability to respond in mind and deed to changing (climatic) conditions (Dercon 2009). Adaptation and poverty alleviation are, however, complex processes embedded in layers of social relations and decision-making power (De Haan and Zoomers 2005). If farmers depend on a degrading natural environment they will have to adapt and adjust agricultural practices to recurring droughts, flood, and other impacts (World Bank 2012). But the ability to adapt in terms of avoiding, controlling or coping with climate change in relation to resources is differentiated, especially where infrastructure is deficient (Klein et al. 2014). Governments, local authorities, and other relevant organisations must therefore facilitate transitions to more sustainable livelihood activities, practices, and strategies (Adhikari 2013).

To study resource access means studying power to control resources once they are accessed. While some have direct control over resources ‘others must maintain their access only through those who have control’. Hence, people are positioned differently as regards power over resources (Ribot and Peluso 2003, p 154). A critical (gender) perspective on property relations and rights to resources that goes beyond access and towards the ability to use and control a particular resource, such as land, would offer a better view than conventional property theory (Ribot and Peluso 2003). Over time and across scales one should study how control relates to existing institutional arrangements, such as the ruling gender regimes of land, labour, production, and reproduction, and how conflict will be handled when it emerges. Existing structures of inequality may reinforce impacts and result in differentiated outcomes. Here some suggest that the use of a continually changing ‘web of access relations’ as a heuristic will allow an analysis of the dynamic processes that influence access to and control over resources (Ribot and Peluso 2003).

### Gendered access to resources—and power to use them

According to some observers, limited access and restricted rights to resources make women particularly vulnerable to climate change (UNDP 2007), especially if crop failure, fuel shortage, and water scarcity affect their capacity to discharge what is deemed to be their food provision responsibility (Kes and Swaminathan 2006). Women and men depend differently on energy, land, water, and other natural and social resources for their everyday life and long-term existence (Doss 2001; Terry 2009; Otzelberger 2011) and it is often stated that women, more than men, face ‘a litany of structural, technological, and cultural barriers’ (Denton 2002, p 17). To fulfil gendered productive and reproductive responsibilities and obligations women may have an interest in, depend on, and manage natural resources while neither having the right to nor being entitled to control these same resources. In many fishing and farming communities, women initiate, participate in and contribute to production while having less (secure) access to natural resources such as land or social resources such as capital, education, and information (Manfre et al. 2013). At the same time, women have multiple reproductive responsibilities and ‘every dawn brings with it a long march in search of fuel, fodder and water’ (Dankelman 2002, p 23).

Based on her extensive review of the literature on women and agricultural production in Africa, Cheryl Doss sees the African farm household as ‘enormously complex’—it is ‘a diversified and multifaceted economic entity’ pursuing numerous varied activities, it operates according to ‘competing goals and objectives’ not least due to gender, and it is involved in ‘elaborate networks of credit, insurance and contracts’ (Doss 2001: 2086–87). The claim that gender is an important analytical category does not, however, prescribe a priori what should be done, according to Doss (2001). Even in cases where gendered rights, roles, and responsibilities are considered, the actual dynamics, practices, and strategies in a certain setting in relation to land, labour, and resources may not be well understood, and interventions including technology adoption designed for women have therefore often failed (Doss 2001).

Nevertheless, economic activity and productive and reproductive responsibilities, even if burdensome, may offer women certain bargaining power (Jabeen 2014) and render them an opportunity to become resourceful agents of change with adaptive capacity (Gabrielsson and Ramasar 2013). Albeit causality is difficult to establish between power and desired outcomes, feminist economists see intra-household bargaining power as a discursive resource that women use to improve decision-making and social position (Doss 2013). And based on the following typology of power, researchers can explore social relations in climate change adaptation in terms of ‘power to’ meaning the ability to adapt, improve or transform; ‘power with’ involving a joint or collective action with other people; ‘power over’ such as challenging and overcoming an instance of social exclusion or subjugation; and ‘power within’ as an individual cognitive process of confidence and consciousness (De Haan and Zoomers 2005). Further, Kabeer (2011) sees empowerment as a specific, structural and path-dependent process influencing ability, capacity, and willingness—in a given place or situation—to accept gender norms and other social constraints or influence, challenge, and change them. She (Kabeer 2011, p 499) speaks of the ability to participate on equal terms with men in reshaping society, the capacity to exercise strategic control over one’s own life, and the willingness to question one’s position in society. Such reasoning is applicable also to vulnerability and climate change adaptation, showing that gender, and changes in gender norms, is both an individual and societal process, both agency and structure.

Scholars who offer constructive advice on increased efficiency and gender equality in agricultural intervention and extension services, in climate change adaptation, and in policy related to both, have suggested a transformative gender approach (Manfre et al. 2013; Farnworth and Colverson 2015). It would not only seek to make gradual improvements to meet women’s (immediate) needs, but also engage with men more profoundly to enable a change in gender relations and the gendered coding of responsibilities in the long term. To achieve that, we would have to use methods that better fit the given agricultural context, reach women and men at times and places that are convenient to their productive and reproductive responsibilities, and acknowledge gender variation in priorities, labour productivity, and time use (Manfre et al. 2013). For policy, researchers suggest that we could address gender relations by following an ‘empowerment pathway’ (Farnworth and Colverson 2015) that actively involves not only women but also men while at the same time rethinking the coding of male and female work tasks (Jerneck and Olsson 2012; Farnworth and Colverson 2015). A similar approach is suggested by scholars who have launched a new and detailed research agenda on this issue for agricultural interventions (Farnworth et al. 2016).

The many inequalities in climate change can be theorised in different ways. If sustainability science puts a feminist political ecology lens on climate change and natural resources management, it may better understand the gendering of impacts, responses, and decision-making power (Andersson 2014). Yet, that necessitates a contextual and intersectional understanding of climate change impacts (Kaijser and Kronsell 2014) and presupposes a comprehensive view of gendered subjectivities and identities (Sultana 2014).

## Designing gender-sensitive research on adaptation: what is the agenda?

Every single research account is necessarily selective, partial, and incomplete (Stoecker 1991). How we pose research questions and construct data will thus affect how we perceive, interpret, and act upon results (O’Neill et al. 2010). The choices I have made here in terms of formulating three guiding questions inspired by Barnett (2010) as well as defining core concepts and identifying the substance of the analysis have resulted in a particular account. Bearing this in mind, I suggest a frame with questions for how one might proceed in gender-sensitive sustainability science research on climate change adaptation in the global South, which uncovers how individual agency is structured by higher order processes in society—such as class and gender. To that end, the dynamics of gender relations, positions, and norms will be core, not the least in how such institutions are challenged by global-to-local environmental pressures on land, water, and other resources.

### Asking gender-sensitive questions

If gender plays out differently in different settings and must be understood ‘in all its local variations’ it still entails sufficient internal coherence to be seen as a whole—although a differentiated whole (Gunnarsson 2011, p 34). Starting from that perspective the specific objective in sustainability science research on climate change adaptation could be to study place-based perceptions of, experiences from, and responses to climate change impacts and risk. In more detail, we could study how farmer’s activities, priorities, skills, and decisions relating to climate change adaptation interact with gender norms in livelihood production and reproduction. Further, we could study how the gendered capacity to make informed decisions on actions aimed at climate change adaptation is manifested, especially if some adaptation strategies contribute to, while others undermine, long-term principles of sustainability.

In concrete terms, it has recently been argued that climate change adaptation should include a gender-sensitive analysis while climate actions require gender sensitivity *strategies* (Alston 2013). Here a comparative analysis across sites and scales may bring comprehensive and more meaningful understandings that can inform policies and programmes—if and how climate change will impact the lives of women and men differently (Sultana 2014; Alston 2013). To avoid preserving or reinforcing gender inequalities, it is essential that both politics and research on the grounded realities of climate events and climate change adaptation should be informed by social justice and gender-sensitive approaches (Sultana 2014). Hence, feminists argue that theory and practice in climate change adaptation and disaster management should communicate more with each other on common issues such as gender justice and the reduction of environmental risks (Enarson 2013) while also observing the agenda on global justice in the development debate (Cornwall and Rivas 2015).

Findings from in-depth studies on the dynamics of gender regimes for land, labour, natural resources, and power (Steen 2011) can be used to frame further research. As regards the social construction of gender and its manifestations in a particular setting or situation, it is helpful to distinguish systematically between three dimensions—the abstract, the concrete social or collective, and the concrete individual (Franz-Balsen 2014). Comparing concrete and specific situations with abstract and general thinking means comparing what is in flux with what is fixed, thus comparing the dynamic process of reality (flux)—such as potentially changing gender norms under socio-ecological stress—with static concepts (fixed)—such as those found in theory (Gunnarsson 2011, p 34). In case of tension between the two, new ideas and concepts can be discovered, formulated, and proposed—and action taken.

One way to explore adaptation is to be informed not only by theories on gender and social power (De Haan and Zoomers 2005) or transformative power (Avelino and Rotmans 2009), but also by discursive institutionalism offering tools to identify early signs of social change as expressed in the (not so easily observable) thinking and speaking that precedes women’s and men’s more observable acting (Schmidt 2008). Tracing, scrutinising, and challenging discursive constructions is thus an important aspect of feminist research, also on climate change (MacGregor 2010). Based on that, and as guidance in research, I pose nine descriptive, interpretive, explanatory, and potentially prescriptive questions:

- How is gender addressed and described in policy documents?
- How are small-scale farmers, women, and men, represented in policy documents?
- How do small-scale farmers, women, and men, perceive risks relating to climate change?
- How do small-scale farmers, women, and men, experience and act upon climate change?
- How does gender affect the adoption/non-adoption of a certain practice or strategy?
- Under what conditions do women/men share or take over women’s/men’s work?
- How do adaptation activities challenge existing gender norms, relations, and dynamics?
- Which contradictions, paradoxes, and tensions contribute to reconstruct gender relations?
- How can gender-sensitive findings inform climate adaptation politics and interventions?

### Seeking general patterns with local variation

Before adaptation strategies are introduced or implemented it is crucial to recognise the complexity, dynamics, and specificity of the setting. Every adaptation initiative and intervention will thus have to be place-sensitive and designed to fit a particular context, in itself imbued with special environmental features, cultural values, and social structures (Moser 2014). But general lessons are still useful. Even if it is not necessarily productive to seek universal remedies across communities or regions it is worthwhile to seek overall patterns that allow scope for certain contextual variation. In line with that, insights on gender dynamics of climate change adaptation can be gained from conducting a critical case where findings are extended analytically beyond a particular setting (Burawoy 2009). The case could concern the gender dynamics of climate change adaptation in comparable sub-Saharan Africa regions (and communities) expected to be hit by more frequent and severe droughts (Dirkx et al. 2008) and could draw on the questions above.

The complexity of interacting factors makes the extended case method an appropriate strategy for this type of research (Burawoy 1998, 2009). It suggests that in-depth studies are theorised and contextualised in relation to broader social dynamics, such as climate change, and higher order processes in society, such as gender. The extended case method can help fulfil the intention of expanding empirical and theoretical findings beyond the scope of the actual research site, *and* demonstrate the strength of up-scaling and transferability of local insights into settings of similar conditions.

## Conclusions

This article has discussed three central questions on climate change adaptation in the context of small-scale agriculture in the global South: what is the purpose of adaptation, what are the potential parallels with other processes such as development and technology uptake, and what are the core institutions that sustainability science, policy, and practice must consider?

Starting from a feminist sustainability science perspective on climate change adaptation and by referring to the development discourse and its gendered aspects and institutions, three main findings emerged. The purpose of adaptation is obviously and per definition to respond to climate change impacts, in both a proactive and reactive sense. In the best case scenario adaptation would tackle poverty, inequality, food insecurity, and ill-health simultaneously and synergetically, while continually taking gender into consideration as one defining institution in the context of small-scale agriculture, especially as regards rights, responsibilities, and strategic achievements. Such adaptation would have a transformative potential.

The parallel drawn between adaptation and other gendered processes, such as development, served to compare preconditions for and actual pathways in climate change adaptation with how the development discourse has understood women and gender differently over time and in varied domains and debates including that of women/gender and nature. From development we can learn how the dynamics of gender, environment, and power play out in the context of multiple stressors such as poverty, inequality, food insecurity, and ill-health. From influential development economist we hear that the notion of ‘inclusive pro-poor growth’ is key to social change, but that must be further scrutinised in light of climate change impacts and responses. Some underline that there are ‘strong overlaps between growth policy and adaptation policy’ and that improvements in development indicators are effective in reducing climate change vulnerability, especially if public policy takes a renewed responsibility while collective action gains greater prominence (Bowen et al. 2012, p 103). This is still to be seen. Climate change adaptation is not really a matter of pro-poor policies emerging from the development discourse, but a call for fairer distribution and stronger recognition of rights—in a world of growing inequality and global concentration of wealth (Piketty and Saez 2014).

As regards institutions, gender is contingent and culturally constructed. Gender norms may thus be subject to change during major social processes such as those in focus. Throughout the analysis it was made clear that gender is a critical social category in development and climate change adaptation alike. Not only does it have multiple institutional implications for adaptation and environmental justice, it also enriches the understanding of climate change impacts and responses—and how sustainability science as an umbrella field could tackle this.
